# Does the Reading of Different Orthographies Produce Distinct Brain Activity Patterns? An ERP Study

**DOI:** 10.1371/journal.pone.0036030

**Published:** 2012-05-15

**Authors:** Irit Bar-Kochva, Zvia Breznitz

**Affiliations:** Department of Learning Disabilities, Edmond J. Safra Brain Research Center for the Study of Learning Disabilities, University of Haifa, Haifa, Israel; Cuban Neuroscience Center, Cuba

## Abstract

Orthographies vary in the degree of transparency of spelling-sound correspondence. These range from shallow orthographies with transparent grapheme-phoneme relations, to deep orthographies, in which these relations are opaque. Only a few studies have examined whether orthographic depth is reflected in brain activity. In these studies a between-language design was applied, making it difficult to isolate the aspect of orthographic depth. In the present work this question was examined using a within-subject-and-language investigation. The participants were speakers of Hebrew, as they are skilled in reading two forms of script transcribing the same oral language. One form is the shallow pointed script (with diacritics), and the other is the deep unpointed script (without diacritics). Event-related potentials (ERPs) were recorded while skilled readers carried out a lexical decision task in the two forms of script. A visual non-orthographic task controlled for the visual difference between the scripts (resulting from the addition of diacritics to the pointed script only). At an early visual-perceptual stage of processing (∼165 ms after target onset), the pointed script evoked larger amplitudes with longer latencies than the unpointed script at occipital-temporal sites. However, these effects were not restricted to orthographic processing, and may therefore have reflected, at least in part, the visual load imposed by the diacritics. Nevertheless, the results implied that distinct orthographic processing may have also contributed to these effects. At later stages (∼340 ms after target onset) the unpointed script elicited larger amplitudes than the pointed one with earlier latencies. As this latency has been linked to orthographic-linguistic processing and to the classification of stimuli, it is suggested that these differences are associated with distinct lexical processing of a shallow and a deep orthography.

## Introduction

Orthographies are conceived on a scale from shallow orthographies with clear grapheme-phoneme correspondence, to deep orthographies, in which these relations are opaque [1]. Many behavioral studies have shown that phonological decoding of small orthographic units is more active in reading shallow orthographies, whereas the direct identification of larger orthographic units is more active in reading deep orthographies [1]–[3]. These differences were suggested to reflect the constraints imposed by the type of script: while simple grapheme-phoneme correspondence is efficient when grapheme-phoneme relations are transparent, it may be insufficient when these relations are opaque.

Less is known about brain activity when reading orthographies of different depths. One of the few studies investigating this subject is the PET study by Paulesu and his colleagues [4]. In this study English readers showed stronger activations than readers of the shallower Italian orthography in the left posterior inferior temporal gyrus and in the anterior inferior frontal gyrus, areas associated with irregular word reading and whole word retrieval. Italians showed stronger activation in left superior temporal regions, associated with phoneme processing. In another study, Simon and his colleagues [5] examined ERPs of French monolinguals and French and Arabic (a language with a deeper orthography than French, when presented without diacritics) bilinguals in a lexical decision task. The N320 component, associated with spelling-to-sound conversion [6], [7], was obtained in both groups when frequent French words and pseudowords were presented, but not when Arabic words were introduced.

A cross-language investigation was applied in these brain-imaging studies – a design in which linguistic differences may be confounded with orthographic depth [8]. When both between-language and between-subject designs are used [4], inter-subject heterogeneity resulting from a variety of socio-cultural differences may also be involved. The study of bilinguals [5] avoids these methodological difficulties but at the same time may introduce cognitive factors specific to bilinguals [9]. A within-subject as well as a within-language study design would contribute to the isolation of orthographic depth when exploring its relations with brain activity. This was the design employed in the present research.

The study of Hebrew readers allows the implementation of such a study design, as two forms of script differing in orthographic depth transcribe the same oral language. The one is the pointed script, in which diacritics, conveying mostly vowel information, are inserted into consonant letters; together, these permit an almost unambiguous conversion of spelling to sound. The second is the unpointed script, which is devoid of diacritics and therefore partially lacks phonemic information, resulting in orthographic opacity.

It should be noted that although readers of Hebrew are skilled in reading both forms of script, they are usually more exposed to the unpointed one. Nevertheless, in previous behavioral studies skilled readers of Hebrew were found to change their everyday reading routine in reading the script they were less exposed to, despite the fact that it was unnecessary. Namely, in several studies larger effects of word-frequency and semantic-priming were obtained in reading unpointed words than in reading pointed words [8], [10], [11]. These results were taken to suggest that the readers used lexical representations in oral reading of the unpointed script. The pointed script was found to enhance oral reading when compared to the unpointed one [8], [10]–[12], suggesting that phonological extraction of the diacritics was involved. As in these studies frequent non-homographic words were introduced, the pointed words could have been easily identified by skilled readers without the extraction of the phonemic information denoted by the diacritics. The distinction between pointed and unpointed reading appears to be less pronounced in the absence of a demand for pronunciation [8], [10], [11], [13]–[15]. Nonetheless, the application of different experimental manipulations did reveal that when the pointed script is presented the decoding of the phonological information conveyed by the diacritics necessarily takes place [15], [16]. These findings imply that the reading routine of Hebrew readers was determined by the type of script presented, rather than by the extent of exposure to each form of script. As previously suggested, if readers of Hebrew prefer to change their everyday reading routine, even in reading frequent non-homographic words, then readers of other shallow and deep orthographies would, all the more so, apply distinct reading routines [8]. Therefore, the study of Hebrew readers may provide important insights into the question whether a shallow and a deep orthography produce different brain activity.

In the present study ERP recordings were taken while adult Hebrew readers carried out a lexical decision task with pointed and unpointed words. Predictions were made regarding visual-perceptual and orthographic-linguistic stages of written word processing. Three components were identified at these stages (N170, P2 and P3) and these were statistically analyzed. The N170 component, peaking around 170 ms, characterized by occipital-temporal negativity and central positivity, was found to be the first to distinguish between orthographic strings and other classes of visually presented stimuli [6], [17]–[20]. If the two forms of script direct the reader into different orthographic processing, then distinct brain activity would be expected from this stage on. At the same time, it should also be taken into account that the two forms of script differ not only in orthographic depth but also visually (as the diacritics are attached to the pointed script only). Tarkiainen and his colleagues [21] found that at around 150 ms after target onset larger negative amplitudes at inferior occipital-temporal sites were associated with longer sequences of stimuli, whether these were orthographic or non-orthographic. This may indicate that the N170 component is sensitive to visual load, and that this effect is not restricted to orthographic processing.

In an attempt to disentangle the possible effects of the visual and the orthographic differences between the two forms of script on early visual-perceptual processing, another visual decision task was administered in which non-orthographic stimuli were presented with or without invented diacritics (Table S1). Participants were asked to make a decision regarding the orientation of these stimuli. If the visual difference between the two forms of script interacts with their processing, then similar differences in the N170 component would be expected when stimuli with and without diacritics, orthographic or non-orthographic, are compared. However, if the two forms of script elicit distinguishable brain activity reflecting distinct orthographic processing from this early stage, then a different pattern of results would be expected when orthographic and non-orthographic stimuli with and without diacritics are compared.

Further differences in brain activity would be expected later on if, in line with previous behavioral studies, orthographic depth directs the readers into different orthographic-linguistic processing leading to lexical identification. Such aspects of processing may be revealed from around 250–300 ms after target onset, although they may begin much earlier [6], [22]–[25]. The two amplitudes analyzed at these later latencies were the positively peaking P2 and P3, found in various tasks involving stimuli discrimination, including lexical decision [26]–[30].

In summary, the aim of the present work was to examine whether the reading of scripts of different orthographic depths induces distinct brain activity. To this end, a within-subject-and-language study design was applied, in which electrophysiological responses were recorded from readers of the shallow and the deep forms of Hebrew orthography.

## Results

### Electrophysiological Measures

**Figure 1:**
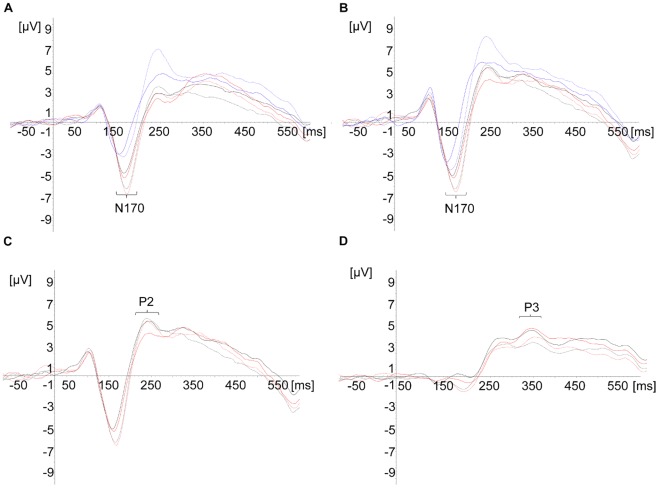
N170, P2 and P3 amplitudes elicited by the different stimuli. A. N170 amplitude at a left occipital-temporal electrode (PO7). B. N170 amplitude at a right occipital-temporal electrode (PO8). C. P2 amplitude at a right occipital-temporal electrode (PO8). D. P3 amplitude at a central-parietal electrode cluster (PO3, PO4 and POZ). Continuous lines represent stimuli without diacritics and dashed lines represent stimuli with diacritics. Words are colored black, pseudowords red, and sequences of squares are in blue.

**Figure 2:**
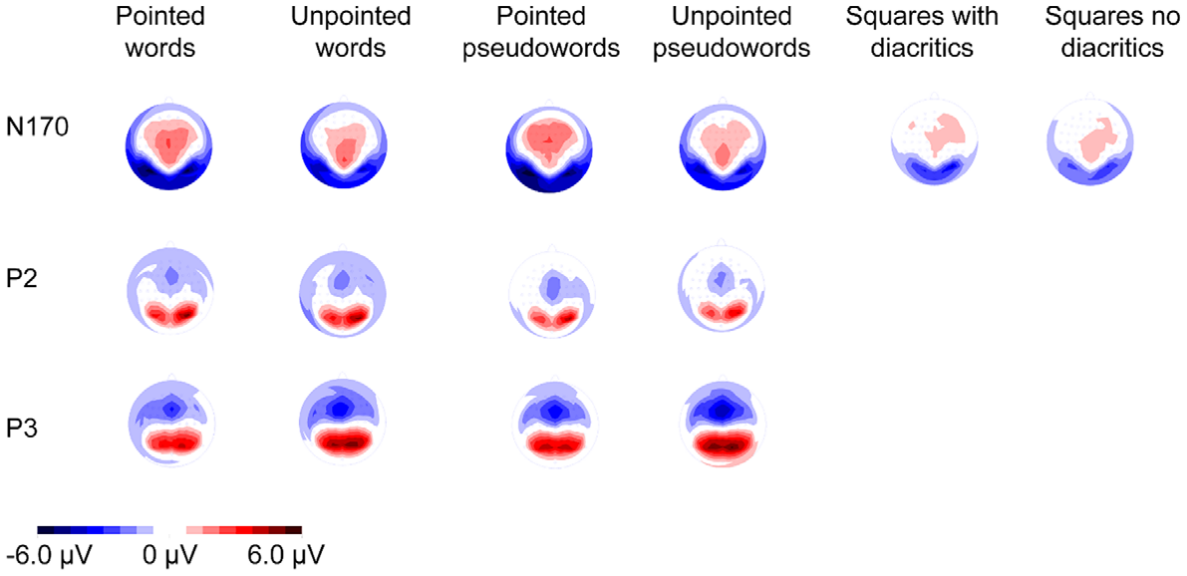
Scalp topographies of the N170, P2 and P3 amplitudes elicited by the different stimuli. Scalp topographies in response to the presentation of words, pseudowords (N170, P2 and P3) and squares (N170) with and without diacritics. Red represents positive electrophysiological activity and blue represents negative electrophysiological activity.

The global field power based on all channels [31] and scalp distributions of each participant and across participants (the grand averages) were first visually inspected. Three time-windows indicated distinct brain activity in response to the presentation of the different stimuli: 120–180 ms, 220–280 ms and 320–380 ms. Within each of these time-windows, stimuli with and without diacritics showed similar topographies, while differences were observed in amplitudes and latencies at the electrodes showing maximum activity (Fig. 1–2). Presuming the recurring pattern of activity at these sites and latencies reflected brain activity associated with the processing of the stimuli presented, these were selected for statistical analysis [31]. In order to reduce bias associated with peak detection of a single point on an amplitude, the amplitudes’ strength was calculated as the mean activity recorded during 25 ms around the peaks observed.

#### 120–180 ms (N170)

The possible effect of the visual load imposed by the Hebrew diacritics on early brain activity was evaluated by analyzing the lexical decision and the non-orthographic orientation decision tasks in one Repeated Measure ANOVA analysis. Similar negative maximums were evoked at this latency by the different stimuli at left and right occipital-temporal electrodes PO7 and PO8. Therefore, a 2×3×2 Repeated Measure ANOVA was carried out, with electrode (PO7/PO8), stimulus (words/pseudowords/squares) and diacritics (with/without meaningful or meaningless diacritics) as within-subject factors.

Amplitudes (Table 1): Main effects of stimulus (*F*_(1.12,34.80)_ = 9.70, *p*<.01, *η_p_^2^* = .24) and diacritics (*F*_(1,31)_ = 52.92, *p*<.001, *η_p_^2^* = .63) were obtained. Bonferroni pair-wise comparisons indicated that words and pseudowords evoked larger amplitudes than sequences of squares (*p*<.05 and *p*<.01, respectively). The means showed that stimuli with diacritics elicited larger amplitudes than stimuli without diacritics.

**Table 1:** Mean amplitudes (in µV) of the components analyzed (standard deviations in parentheses).

Components	Electrodes	Pointed words	Unpointedwords	Pointedpseudowords	Unpointed pseudowords	Squares withdiacritics	Squares without diacritics
N170	PO7	−6.45 (5.15)	−4.92 (4.61)	−6.61 (5.06)	−5.38 (4.85)	−4.07 (3.83)	−3.56 (3.98)
	PO8	−6.46 (5.51)	−5.16 (5.13)	−6.72 (5.22)	−5.44 (4.99)	−4.75 (5.86)	−3.53 (5.65)
P2	PO8	6.31 (4.68)	6.23 (4.61)	5.87 (5.50)	5.29 (4.35)	–	–
P3	Average of PO3,PO4, POZ	4.33 (4.25)	5.36 (3.88)	4.87 (4.54)	5.73 (3.91)	–	–

Latencies (Table 2): Main effects of electrode (*F*_(1,31)_ = 8.99, *p*<.01, *η_p_^2^* = .22), stimulus (*F*_(1.30,40.36)_ = 13.60, *p*<.001,*η_p_^2^* = .30) and diacritics (*F*_(1,31)_ = 26.72, *p*<.001, *η_p_^2^* = .46) were obtained, in addition to an interaction between these three variables (*F*_(2,62)_ = 3.62, *p*<.05, *η_p_^2^* = .10). Bonferroni pair-wise comparisons showed that words and pseudoword elicited delayed amplitudes in comparison to squares (*p*<.01, *p*≤.001, respectively). The mean latencies indicated that stimuli with diacritics elicited delayed amplitudes in comparison to stimuli without diacritics, and that amplitudes were evoked earlier at right electrode PO8 than at left electrode PO7. The means also suggested that the direction of differences between stimuli with and without diacritics was similar in both electrodes. At the same time, planned comparisons showed that while the differences between pointed and unpointed words and between squares with and without diacritics were significant at both electrode PO7 (*t*_(31)_ = 2.67, *p*<.05, *t*_(31)_ = 2.82, *p*<.01, respectively) and PO8 (*t*_(31)_ = 3.46, *p*<.01, *t*_(31)_ = 5.90, *p*<.001, respectively), the difference between pointed and unpointed pseudowords was significant at PO7 (*t*_(31)_ = 3.20, *p*<.01), but not at PO8 (*t*_(31)_ = 1.44, *p* = .16).

**Table 2:** Mean latencies (in ms) of the components analyzed (standard deviations in parentheses).

Components	Electrodes	Pointed words	Unpointedwords	Pointedpseudowords	Unpointed pseudowords	Squares withdiacritics	Squares without diacritics
N170	PO7	170.13 (11.15)	164.78 (13.55)	171. 89 (10.25)	166.06 (12.83)	160.19 (15.48)	157.07 (14.85)
	PO8	167.62 (11.67)	162.69 (11.99)	166.88 (13.98)	164.02 (11.39)	159.55 (13.61)	153.44 (13.23)
P2	PO8	251.60 (22.34)	253.68 (25.11)	255.55 (22.43)	253.80 (24.43)	–	–
P3	Average of PO3,PO4, POZ	341.11 (23.63)	338.56 (19.94)	348.71 (24.04)	340.01 (21.67)	–	–

#### 220–280 ms (P2)

From this time-window on the data from the non-orthographic orientation decision task were excluded from analysis, as beyond the early stages of visual perception this task and an orthographic-linguistic task may impose essentially different demands of processing. A maximum positive activity was observed at the right occipital-temporal electrode PO8. A 2×2 Repeated Measure ANOVA was carried out, with stimulus (words/pseudowords) and form of script (pointed/unpointed) as within-subject factors.

Amplitudes (Table 1): A main effect was found for stimulus (*F*_(1,31)_ *= *7.83, *p*<.01, *η_p_^2^* = .20), with larger amplitudes evoked by words than by pseudowords. Although no interaction between stimulus and form of script was found, the means suggested larger differences between unpointed words and unpointed pseudowords than between pointed words and pointed pseudowords. Planned comparisons indicated that only the difference between the unpointed stimuli was significant (*t*_(31)_ = 2.36, *p*<.05).

Latencies (Table 2): No significant effects were obtained.

#### 320–380 ms (P3)

Maximum positive activity was observed at 3 adjacent central-parietal electrodes: PO3, PO4 and POZ. A 2×2 Repeated Measure ANOVA with stimulus (words/pseudowords) and form of script (pointed/unpointed) as within-subject factors was conducted on the mean amplitudes and latencies of this cluster of electrodes.

Amplitudes (Table 1): A main effect of form of script was obtained (*F*_(1,31)_ = 15.96, *p*<.001, *η_p_^2^* = .34), with unpointed stimuli evoking larger amplitudes than pointed stimuli. The main effect of stimulus approached significance (*F_(_*_1,31*)*_ *= *3.50, *p = *.07, *η_p_^2^ = *.10). The mean amplitude of pseudowords tended to be larger than the mean amplitude of words. The interaction between stimulus and form of script was insignificant. At the same time, planned comparisons indicated that pointed words and pointed pseudowords marginally differed in amplitudes (*t*_(31)_ = −2.04, *p≤*.05), while the difference between unpointed words and unpointed pseudowords was no longer significant.

Latencies (Table 2): The main effect of form of script was significant (*F*_(1,31)_ = 5.65, *p*<.05, *η_p_^2^* = .15), with earlier amplitudes evoked by unpointed stimuli than by pointed ones. The main effect of stimulus approached significance (*F*_(1,31)_ = 3.48, *p* = .07, *η_p_^2^* = .10), suggesting that words tended to evoke earlier amplitudes than pseudowords.

### Experimental Behavioral Measures

A 2×2 Repeated Measure ANOVA with stimulus (words/pseudowords) and form of script (pointed/unpointed) as within-subject factors was conducted on the measures of accuracy and reaction times of the lexical decision task (Table 3).The participants reached ceiling accuracy in all conditions. With regard to reaction times, a main effect of stimulus was found (*F*_(1,31)_ = 41.61, *p*<.001, η_p_^2^ = .57) with words identified faster than pseudowords. A main effect of form of script was also obtained (*F*_(1,31)_ = 8.01, *p*<.01, η_p_^2^ = .20), with longer reaction times for pointed stimuli than for unpointed stimuli. The interaction between type of stimulus and form of script was marginally significant (*F*_(1,31)_ = 4.08, *p≤*.05, η_p_^2^ = .12). *t*-tests suggested significantly longer reaction times in response to the pointed script only in reading of words (*t*_(31)_ = 3.29, *p*<.01). Also, the difference between unpointed words and unpointed pseudowords was larger (*t*_(31)_ = −6.91, *p*<.001) than the difference between pointed words and pointed pseudowords (*t*_(31)_ = −3.28, *p*<.01). Notably, ceiling accuracy was also obtained in the non-orthographic task (Table 3). Planned *t-*test comparisons between the non-orthographic stimuli (straight squares only) with and without diacritics showed no significant difference in reaction times (*t*_(31)_ = 1.69, *p* = .10).

**Table 3:** Mean accuracy (in percentages) and reaction times (in ms) per condition (standard deviations in parentheses).

	Lexical decision			
	Pointed		Unpointed	
	Words	Pseudowords	Words	Pseudowords
RT	669.01 (110.26)	699.48 (102.82)	630.46 (82.54)	683.27 (92.86)
Accuracy	95.68 (4.33)	96.59 (5.30)	96.25 (3.72)	95.63 (6.63)
	**Non-orthographic orientation decision (squares)**			
	**With diacritics**		**Without diacritics**	
	**Straight**	**Tilted**	**Straight**	**Tilted**
RT	512.40 (78.18)	–	501.17 (62.96)	–
Accuracy	97.96 (1.98 )	–	98.47 (1.71)	–

## Discussion

The two forms of script induced distinct brain activity patterns at early and late stages of processing. At an early stage stimuli with diacritics, whether orthographic or non-orthographic, elicited larger N170 amplitudes with delayed latencies than stimuli lacking diacritics at occipital-temporal sites. These results accord with the aforementioned study by Tarkiainen et al. [21], who found a similar early effect of the physical properties (the length) of visual orthographic and non-orthographic stimuli on brain activity. Therefore, the diacritics may have loaded on early visual-perceptual processing. It appears then that the difference in early brain activity in response to the presentation of pointed and unpointed orthographic stimuli can, at least partially, be explained by the different visual-spatial appearance of the two forms of script.

Nevertheless, differences in early orthographic aspects of processing may also account for the results obtained. The smaller N170 amplitudes elicited by sequences of squares than by words and pseudowords are in line with previous studies showing this component to be the first to distinguish between orthographic and non-orthographic processing [6], [17], [19], [20]. As the process of transcribing the diacritics to their corresponding phonemes was found to be an automatic process for adult readers of Hebrew [12], [15], [32], [33], it is possible that such a process had begun early on in reading the pointed script. Some indication of such early phonological processing in reading shallow orthographies around the same time window can be found in other ERP studies [18], [19], [23], [24].

As readers of Hebrew are usually more exposed to unpointed texts than to pointed texts, another possibility is that pointed and unpointed words differed in terms of visual-orthographic familiarity. However, the N170 amplitudes of words (familiar orthographic pattern) and pseudowords (unfamiliar orthographic pattern) within each form of script did not differ. Moreover, the literature does not provide consistent evidence for an effect of familiarity with the word-form on the N170 component, even under conditions controlling for possible effects of lexicality [24], [34], [35].

At later stages of processing, at around 250 ms after target onset, words elicited larger amplitudes than pseudowords at a right occipital-temporal site, with no significant interaction with form of script. This difference may be a consequence of the type of decision made (recognizing meaningful words and rejecting meaningless words). At the same time, this timing was associated with orthographic-linguistic stages of written word processing [6], [22]. Therefore, this effect suggests that some level of lexical processing had begun no later than 250 ms after target onset in reading both forms of script.

An effect of form of script was obtained later on, at around 340 ms, with unpointed orthographic stimuli eliciting larger amplitudes with earlier latencies in comparison to pointed orthographic stimuli at central-parietal sites. As the general morphology and topography of the brain activity elicited by pointed and unpointed stimuli were very much alike while the strength of the amplitudes differed, it may be that stimuli presented in both forms of script were similarly processed but required a different intensity of processing. The latencies and topographies of the amplitudes elicited around 340 ms resembled the well documented P3 component, found in tasks involving stimuli discrimination [26]–[29]. The P3 amplitude has been linked to the amount of attention engaged in the task [26]. A difficult or a complex task is expected to impose more demands on attention resources and, as a result, to induce a larger P3 amplitude. Considering the visual load imposed by the diacritics at an earlier stage of processing, in addition to the fact that the readers were less exposed to pointed texts than to unpointed ones, one would expect an enhanced P3 amplitude in pointed reading rather than in unpointed reading. In view of the opposite pattern of results obtained, this interpretation seems less likely.

An alternative explanation would be that pointed and unpointed orthographic stimuli were processed differently. It may be that, in line with behavioral studies of reading shallow and deep orthographies, the phonologically-mediated route was more active in recognizing pointed words than unpointed words, while the direct lexical route was more active in recognizing unpointed words [1], [2]. According to connectionist models of reading, the phonologically-mediated access to the lexicon is slower than the direct access, as the first involves more steps [36]. Although no interactions between stimulus and form of script were obtained, planned comparisons did suggest that the amplitude of words differed from that of pseudowords earlier when stimuli were presented in the unpointed script (250 ms) than in the pointed script (340 ms). These results may suggest some difference in the timing of lexicality effects in reading the two forms of script. It may also be noted, that if the smaller P3 amplitude elicited by the pointed script than by the unpointed one reflected reduced effort invested in the task [37], then these results may shed light on previous findings suggesting the phonologically-mediated procedure to be the preferred, or the default, routine of word identification [1], [8].

At the same time, attenuating effects on the amplitudes of the pointed script must also be considered. Such effects may be derived from models conceptualizing the factors underlying the amplitude of P3. Weak matches between a perceived stimulus and its internal representation, load imposed on working memory, as well as limited practice were all suggested to have an attenuating effect on the P3 amplitude [26], [38]. Bearing in mind that the participants were more accustomed to reading the unpointed script than the pointed one, such attenuating effects may have reduced the P3 amplitude for pointed orthographic stimuli. However, the models conceptualizing these factors underlying the amplitude of P3 were based on experiments involving a variety of paradigms, and in some cases also a complex experimental structure from which the exact factors affecting the P3 amplitude could only be assumed [26].

As far as reaction is concerned, the behavioral results showed delayed word recognition time in reading the pointed script compared to the unpointed one. These findings are in conflict with most studies indicating similar reaction times for pointed and unpointed Hebrew words in lexical decision [8], [10], [11], [13], [15]. The reason for these discrepant findings may lie in the different presentation durations of the stimuli. The previous studies either gave no fixed presentation time or utilized a 1000 ms presentation time, while our study used a short presentation duration of 400 ms. Our results do agree, however, with those by Koriat [39], who also found a negative effect of the diacritics on word identification when stimuli were briefly (100–200 ms) projected. Therefore, the pointed script may have a delaying effect on reaction time only under short presentation durations. The visual load imposed by the diacritics at an early stage of visual-perceptual processing, as was found in the present study, may play some role in this negative effect.

A reservation should be made regarding the ecological value of the reading task applied, as the inclusion of pseudowords in a reading task may direct the readers into some level of proofreading which does not reflect natural demands of everyday reading. However, if the pseudowords had directed the readers into such a strategy of reading, pointed and unpointed reading should have been similarly affected. Therefore, such an effect should have attenuated differences in the processing of the two forms of script. The findings indicating differences in brain activity evoked by the two forms of script were, nevertheless, significant.

In conclusion, while further research is required in order to better understand the sources of the distinct brain activity obtained in reading the two forms of script, the results of this within-subject-and-language investigation are in agreement with findings of the previous cross-language studies [4], [5] mentioned in the introduction, and together these suggest that orthographies of different depths induce distinct brain activity. These brain imaging results converge with previous behavioral studies indicating distinct cognitive processing of shallow and deep orthographies [1]–[3], [15].

## Materials and Methods

### Ethics Statement

The participants gave their written informed consent to take part in the study, which was approved by the institutional review board.

### Participants

Thirty-two participants (16 men) took part in the study (age range 20–33 years, mean = 25.93, *SD* = 3.07). All were native speakers of Hebrew, right handed, with normal to corrected vision. Participants reporting no history of reading difficulties, attention disorder or any other learning difficulty or neurological condition were invited to participate in the study. Their general ability and reading skills were examined first, and only those who exhibited no special difficulty in these tests (Table S2) were summoned for another session, during which the experimental tasks were administered.

### Background Measures

#### General ability

The following sub-tests from the WAIS-III [40] were administered: Block Design, Similarities, Symbol Search, Digit-Symbol, Digit Span and Letter-Number Sequencing.

#### Reading

Oral deciphering of pointed consonants and vowels [41]: The participants’ knowledge of the Hebrew diacritics was tested by presenting 42 pointed consonants and vowels which they were required to pronounce.

Oral reading of unpointed words [42]: A list of 168 unpointed words (2–7 letters in length) of different frequencies was presented and the participants were asked to accurately read as many words as possible in 1 minute.

Oral reading of pointed pseudowords [42]: A list of 86 pointed pseudowords arranged in order of increasing length (3–7 letters) was presented. The testing procedure was the same as in the word reading test.

Oral reading of unpointed text (The Center for Psychometric Tests, 1994): A text comprising 216 words was presented to the participants, who were asked to read quickly and accurately.

### Experimental Tasks

The participants completed two computerized visual decision tasks presented using the E-Prime software [43]. The one was a lexical decision task and the other a non-orthographic orientation decision task (examples of the stimuli are presented in Table S1).

#### Lexical decision

Words and pseudowords were presented to the center of the screen (font David 28), and the participants were asked to categorize each stimulus as a meaningful or a meaningless word. Due to the sensitivity of ERP recordings, only certain categories of words were included: the words were non-homographic concrete nouns, 3–5 letters in length, with a single meaning. In addition, none of the words contained the Hebrew vowel letters ? (“*yud*”) and ?(“*vav*”), since in Hebrew pointed script, some of these letters are omitted and replaced by diacritics. The inclusion of such words would have created differences in the number of letters in a word when presented with or without diacritics.

A list of frequent words was compiled mainly out of The Word-Frequency Database for Printed Hebrew [44], a corpus containing the frequency of appearance of words in Israeli newspaper texts. In order to collect a sufficient number of stimuli conforming to the category of words suitable for the study, the words chosen ranged from medium-frequent to very frequent (above 20 occurrences in one million), and additional words were collected from other sources. The first was a corpus of the basic words used in elementary schools [45], whose frequency value was above 9 occurrences in 200,000. The second was a word frequency survey administered to university students, who were asked to estimate the frequency of words according to a scale from 1 (very infrequent) to 7 (very frequent). An average frequency was calculated for each word, and words with an average rating of above 3 were included in the study.

Owing to a considerable amount of variation in the frequency values of the words chosen from each of the three sources, the words were organized in pairs, matched for frequency values based on the same word-frequency database. Words classified according to the word frequency survey were matched also for the number of raters (n = 29 to 50) and percent of agreement between raters. The pairs were also matched for the number of letters in each word. The words in each pair were then split between two parallel lexical decision tasks. Each task was presented in the pointed or the unpointed form of script, in a counterbalanced manner between the participants. Pseudowords were created on the basis of the real words by changing one letter of each word while maintaining the word’s morphological pattern [46], [47]. Each lexical decision task contained 55 words and 55 pseudowords.

### Non-Orthographic Orientation Decision

The two forms of script share the same letters, while diacritics are attached to the letters in the pointed script only. In order to isolate the visual difference between the two forms of script, a non-orthographic task was designed in which the same sequence of non-orthographic stimuli were presented with or without meaningless diacritics. These stimuli were simple sequences of squares (appearing in the place of letters) in order to avoid any visual load not resulting from the diacritics. The length of each sequence and its size were matched to the orthographic stimuli in the lexical decision task. The participants were asked to decide whether the sequences of squares were tilted or not. As this task was used only for the purpose of isolating the visual difference between the two forms of script, and in order to reduce the number of variables, only data on straight squares with and without diacritics was analyzed.

### Procedure

#### Task administration

ERPs were recorded while participants carried out the two decision tasks in a sound-attenuated room. The stimuli were projected at random to the center of the computer monitor for 400 ms. This duration was based on findings indicating that gaze duration on words among adult readers of Hebrew varies from 229 to 267 ms [48], and that lexical access progresses gradually, requiring around 300 ms [25]. Some leeway was added to take into account variation between participants. Another 1600 ms were given to respond.

Eight example trials preceded each task. In order to ensure the participants understood the categorization instruction (word/pseudoword in the lexical decision task and straight/tilted squares in the non-orthographic task) they had to reach an accuracy rate of 70% in these trials in order to proceed to the task (lower accuracy rates resulted in repeated administration of the example trials). The participants were asked to respond immediately after the presentation of each stimulus by pressing with their right hand one keyboard button for words (or straight squares), and another button for pseudowords (or tilted squares).

In order to avoid the adaptation of a default reading strategy that is suitable for both pointed and unpointed reading [39], pointed and unpointed orthographic stimuli (as well as squares with and without diacritics) were presented in separate blocks. The addition of diacritics, as well as the order of the tasks, were counterbalanced between participants.

### EEG Recording and Offline Analysis

Scalp EEG data was continuously recorded using a 64 channel BioSemi ActiveTwo system (BioSemi, Amsterdam, The Netherlands) and the ActiveView recording software. Pin-type electrodes were mounted on a customized Biosemi head-cap, arranged according to the extended 10–20 system. Two flat electrodes were placed on the sides of the eyes to monitor horizontal eye movements. A third flat electrode was placed underneath the left eye to monitor vertical eye movements and blinks. During the session, electrode offset was kept below 50 mV. The EEG signals were amplified and digitized with a 24 bit AD converter. A sampling rate of 2048 Hz (0.5 ms time resolution) was employed.

ERPs were analyzed offline using the Brain Vision Analyzer software (Brain-Products). The EEG data were filtered (high: 25 Hz and low: 0.1 Hz), and referenced to the common average of all electrodes. Ocular artifacts were corrected as described previously [49]. Correct responses were divided into epochs of 100 ms pre-stimulus baseline and 1900 ms post-stimulus. Artifacts were rejected, the resulting data was baseline-corrected, and global field power – RMS [31] was calculated.

## Supporting Information

Table S1 **Examples of the stimuli presented in the lexical decision and the non-orthographic orientation decision tasks.**
(TIF)

Table S2 **General ability and reading scores taken as background measures.**
(DOCX)

## References

[1] Katz L, Frost R, Frost R, Katz L (1992). The reading process is different for different orthographies: The orthographic depth hypothesis.. *Orthography, phonology, morphology, and meaning*.

[2] Frost R, Snowling MJ, Hulme C (2005). Orthographic systems and skilled word recognition processes in reading.. *The science of reading: A handbook*.

[3] Ziegler JC, Goswami U (2005). Reading acquisition, developmental dyslexia, and skilled reading across languages: A psycholinguistic grain size theory.. *Psychol Bull*.

[4] Paulesu E, McCrory E, Fazio F, Menoncello L, Brunswick N (2000). A cultural effect on brain function.. *Nat Neurosci*.

[5] Simon G, Bernard C, Lalonde, R, Rebaï M (2006). Orthographic transparency and grapheme–phoneme conversion: An ERP study in Arabic and French readers.. *Brain Res*.

[6] Bentin S, Mouchetant-Rostaing Y, Giard MH, Echallier JF, Pernier J (1999). ERP manifestations of processing printed words at different psycholinguistic levels: time course and scalp distribution.. *J Cogn Neurosci*.

[7] Simon G, Bernard C, Largy P, Lalonde R, Rebaï M (2004). Chronometry of visual word recognition during passive and lexical decision tasks: An ERP investigation.. *Int J Neurosci*.

[8] Frost R (1994). Prelexical and postlexical strategies in reading: Evidence from a deep and shallow orthography.. *Exp Psychol Learn Mem Cogn*.

[9] Kovelman I, Baker SA, Petitto LA (2008). Bilingual and monolingual brains compared: a functional magnetic resonance imaging investigation of syntactic processing and a possible “neural signature” of Bilingualism.. *J Cogn Neurosci,*.

[10] Frost R (1995). Phonological computation and missing vowels: mapping lexical involvement in reading.. *Exp Psychol Learn Mem Cogn*.

[11] Koriat A, Bouma H, Bouwhuis DG (1984). Reading without vowels: Lexical access in Hebrew.. *Attention and performance X: Control of language processes*.

[12] Navon D, Shimron J (1981). Does word naming involve grapheme-to-phoneme translation? Evidence from Hebrew.. *Journal of Verbal Learning and Verbal Behavior*.

[13] Bentin S, Frost R (1987). Processing lexical ambiguity and visual word recognition in a deep orthography.. *Mem Cognit*.

[14] Koriat A (1985). Lexical access for low and high frequency words in Hebrew.. *Mem Cognit*.

[15] Shimron J (2006). Reading Hebrew: the language and the psychology of reading it..

[16] Bentin S (1989). Orthography and phonology in lexical decision: evidence from repetition effects at different lags.. *Exp Psychol Learn Mem Cogn*.

[17] Brem S, Lang-Dullenkopf A, Maurer U, Halder P, Bucher K (2005). Neurophysiological signs of rapidly emerging visual expertise for symbol strings.. *Cogn Neurosci*.

[18] Maurer U, Brandeis D, McCandliss BD (2005). Fast, visual specialization for reading in English revealed by the topography of the N170 ERP response.. *Behav Brain Funct*.

[19] Maurer U, Brem S, Bucher K, Brandeis D (2005). Emerging neurophysiological specialization for letter strings.. *J Cogn Neurosci*.

[20] Rossion B, Joyce CA, Cottrell GW, Tarr MJ (2003). Early lateralization and orientation tuning for face, word, and object processing in the visual cortex.. *Neuroimage*.

[21] Tarkiainen A, Helenius P, Hansen PC, Cornelissen PL, Salmelin R (1999). Dynamics of letter string perception in the human occipitotemporal cortex.. *Brain*.

[22] Hauk O, Davis MH, Ford M, Pulvermüller F, Marslen-Wilson WD (2006). The time course of visual word recognition as revealed by linear regression analysis of ERP data.. *NeuroImage*.

[23] Proverbio AM, Zani A (2003). Time course of brain activation during graphemic/phonologic processing in reading: An ERP study.. *Brain Lang*.

[24] Proverbio AM, Vecchi L, Zani A (2004). From orthography to phonetics: ERP measures of grapheme-to phoneme conversion mechanisms in reading.. *J Cogn Neurosci*.

[25] Sereno SC, Rayner K, Posner MI (1998). Establishing a time-line of word recognition: evidence from eye movements and event related potentials.. *NeuroReport*.

[26] Kok A (2001). On the utility of P3 amplitude as a measure of processing capacity.. *Psychophysiology*.

[27] Polich J (2007). Updating P300: An integrative theory of P3a and P3b.. *Clinical Neurophysiology*.

[28] Pritchard WS (1981). Psychophysiology of P300.. *Psychol Bull*.

[29] Verleger R, Jaśkowski P, Wascher E (2005). Evidence for an integrative role of P3b in linking reaction to perception.. *J Psychophysiol*.

[30] Breznitz Z, Misra M (2003). Speed of processing of the visual–orthographic and auditory–phonological systems in adult dyslexics: The contribution of ‘‘asynchrony’’ to word recognition deficits.. *Brain Lang*.

[31] Lehmann D, Skrandies W (1980). Reference-free identification of components of checkerboard–evoked multichannel potential fields.. *Electroencephalogr Clin Neurophysiol,*.

[32] Shimron J, Navon D (1982). The dependence on graphemes and on their translation to phonemes in reading: A developmental perspective.. *Read Res Q*.

[33] Smolka E, Zohar E (2006). Phonological and orthographic visual word recognition in the two cerebral hemispheres: Evidence from Hebrew.. *Cogn Neuropsychol*.

[34] Sauseng P, Bergmann J, Wimmer H (2004). When does the brain register deviances from standard word spellings?–An ERP study.. *Cogn Brain Res*.

[35] Maurer U, Zevin JD, McCandliss BD (2008). Left-lateralized N170 effects of visual expertise in reading: evidence from Japanese syllabic and logographic scripts.. *J Cogn Neurosci*.

[36] Harm MW, Seidenberg MS (2004). Computing the meanings of words in reading: Cooperative division of labor between visual and phonological processes.. *Psychol Rev*.

[37] Luck SJ (2005). *An introduction to the event-related potential technique*..

[38] Johnson R (1986). A triarchic model of P300 amplitude.. *Psychophysiology*.

[39] Koriat A (1985). Lateralization effects in reading pointed and unpointed Hebrew.. *Br J Psychol*.

[40] Wechsler D (1997). *WAIS-III Adult Intelligence Scale*..

[41] Shani M, Lachman D, Shalem Z, Bahat A, Zeiger T (2006). *Alef ad Taf (A-Z) Diagnostic test battery for written language disorders.*.

[42] Shatil E (1997). *One-Minute Test for Word and Pseudowords.*.

[43] Schneider W, Eschman A, Zuccolotto A (2002). *E-Prime user’s guide*..

[44] Frost R, Plaut D (2009). *The word-frequency database for printed Hebrew*..

[45] Balgur R (1968). *The basic word list for elementary schools.*.

[46] Frost R, Forster KI, Deutsch A (1997). What can we learn from the morphology of Hebrew? A masked priming investigation of morphological representation.. *J Exp Psychol Learn Mem Cogn* 23:4.

[47] Frost R, Kugler T, Deutsch A, Forster KI (2005). Orthographic structure versus morphological structure: principles of lexical organization in a given language.. *J Exp Psychol Learn Mem Cogn* 31:6.

[48] Deutsch A, Rayner K (1999). Initial fixation location effects in reading Hebrew words.. *Lang Cogn Process*.

[49] Gratton G, Coles MGH, Donchin E (1983). A new method for off-line removal of ocular artifact.. *Electroen Clin Neuro*.

