# Supplementary figures and images for: Does the Reading of Different Orthographies Produce Distinct Brain Activity Patterns? An ERP Study
Source: PLoS One. 2012 May 15;7(5):e36030. doi: 10.1371/journal.pone.0036030 (PMC3352908; doi:10.1371/journal.pone.0036030)

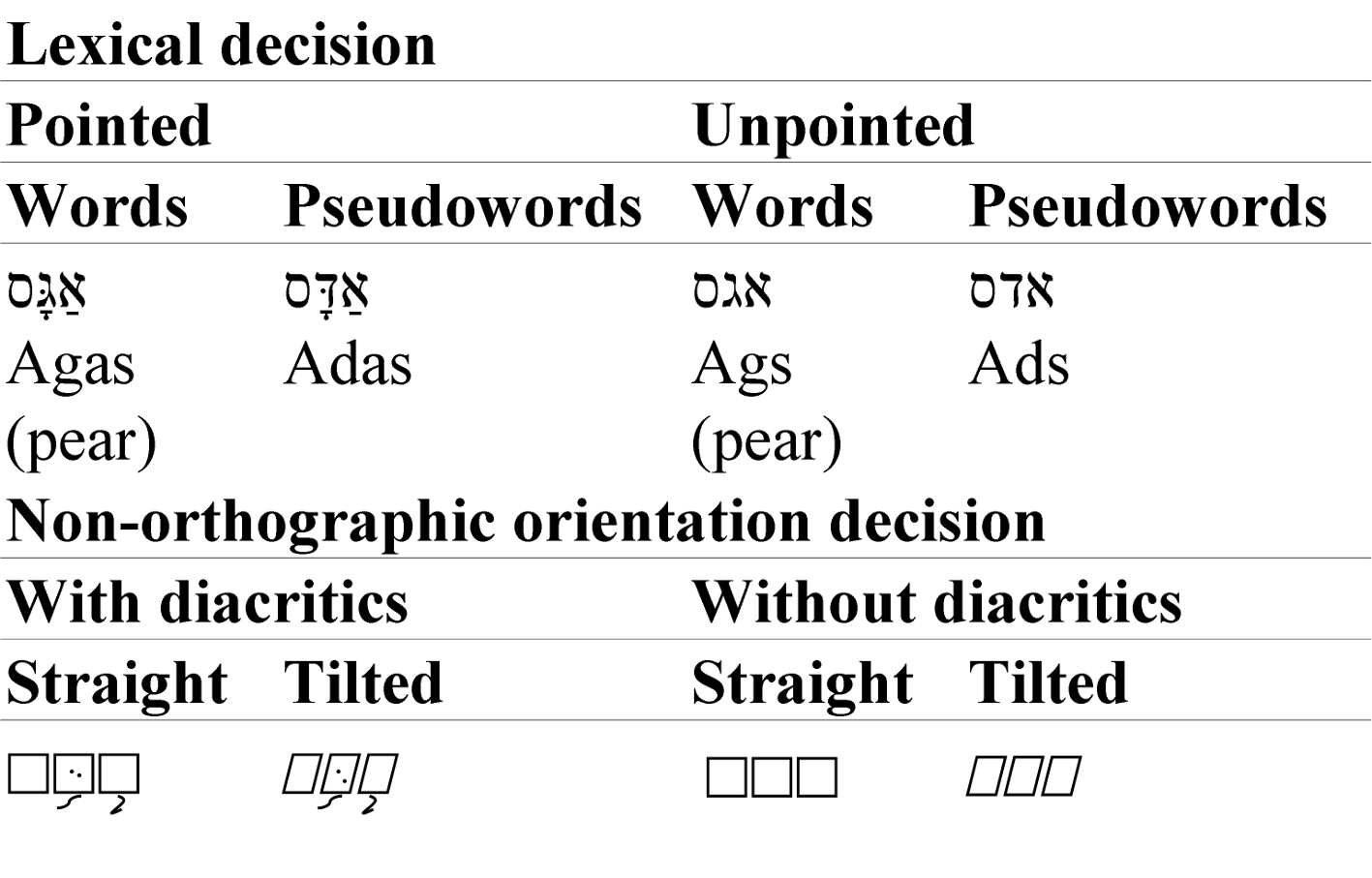

Supplement: Table S1 — Examples of the stimuli presented in the lexical decision and the non-orthographic orientation decision tasks. (TIF) [file pone.0036030.s001.tif]
