# Supplementary material for: Does the Reading of Different Orthographies Produce Distinct Brain Activity Patterns? An ERP Study
Source: PLoS One. 2012 May 15;7(5):e36030. doi: 10.1371/journal.pone.0036030 (PMC3352908; doi:10.1371/journal.pone.0036030)
Supplement: Table S2 — General ability and reading scores taken as background measures. (DOCX) [file pone.0036030.s002.docx]

Table S2. General ability and reading scores taken as background measures.

|  | **Possible range** | **Actual range** | **Mean** | **SD** |
| --- | --- | --- | --- | --- |
| **General ability**  (standard scores) |  |  |  |  |
| Block Design | 1-19 | 7-19 | 12.50 | 2.95 |
| Similarities | 1-19 | 9-18 | 12.47 | 2.58 |
| Digit Symbol | 1-19 | 7-15 | 10.84 | 2.22 |
| Symbol Search | 1-19 | 7-18 | 11.34 | 2.65 |
| Digit Span | 1-19 | 5-19 | 12.31 | 3.26 |
| Letter –Number Sequence | 1-19 | 8-19 | 13.19 | 3.16 |
| **Reading** |  |  |  |  |
| Consonants and Vowels % accuracy | 0-100 | 83-100 | 95.83 | 4.10 |
| Consonants and Vowels output^1^ | - | 45-126 | 86.08 | 21.33 |
| Pseudowords % accuracy | 0-100 | 86-100 | 95.71 | 3.38 |
| Pseudowords output^1^ | - | 37-84 | 65.50 | 11.90 |
| Words % accuracy | 0-100 | 97-100 | 99.45 | .75 |
| Words output^1^ | - | 86-159 | 114.84 | 16.58 |
| Text % accuracy | 0-100 | 98-100 | 99.77 | .42 |
| Text output^1^ | - | 125-240 | 162.53 | 24.12 |

1. Output= correct items read within 1 minute.
